# Effect of Alpha Lipoic Acid Supplementation on Oxidative Stress and Lipid Parameters in Women Diagnosed with Low-Grade Squamous Intraepithelial Lesions (LSILs): A Double-Blind, Randomized, Placebo-Controlled Trial

**DOI:** 10.3390/antiox12091670

**Published:** 2023-08-25

**Authors:** Anja Divković, Zinaida Karasalihović, Ivana Rumora Samarin, Damir Sabitović, Kristina Radić, Nikolina Golub, Lovorka Vujić, Marija Grdić Rajković, Dubravka Vitali Čepo

**Affiliations:** 1 University Clinical Centre Tuzla, 75000 Tuzla, Bosnia and Herzegovina; anja.divkovic1@gmail.com (A.D.); zinaida.karasalihovic@ukctuzla.ba (Z.K.);; 2 University of Zagreb, Faculty of Food Technology and Biotechnology, 10000 Zagreb, Croatia; irumora@pbf.hr; 3 University of Zagreb, Faculty of Pharmacy and Biochemistry, 10000 Zagreb, Croatia; kradic@pharma.hr (K.R.); ngolub@pharma.hr (N.G.); lvujic@pharma.hr (L.V.); mgrdic@pharma.hr (M.G.R.)

**Keywords:** alpha lipoic acid, lipid status parameters, oxidative stress parameters, LSIL, diet quality index

## Abstract

Limited scientific evidence shows that alpha lipoic acid (ALA) can induce regression rates of low-grade squamous intraepithelial lesions (LSILs), but the mechanisms of these effects have not been elucidated. To gain a broader insight into its therapeutic potential and mechanisms of action, the effects of 3 months of supplementation with 600 mg of ALA on antioxidant and lipid status parameters in 100 patients with LSILs were investigated in a randomized, placebo-controlled study. The obtained results are discussed in terms of patients’ initial metabolic status and diet quality (particularly nutritional intake of antioxidants). The obtained results showed that oxidative status biomarkers were not significantly affected by ALA supplementation. However, serum superoxide dismutase (SOD) activity was positively affected in the subgroup of patients with higher dietary antioxidant intake. Surprisingly, ALA supplementation resulted in a small but statistically significant increase in serum low density lipoprotein (LDL), and the observed effect was significantly affected by the initial lipid status of the participants. Larger studies are necessary to gain additional insights on the clinical significance of ALA as an antioxidant and hypolipemic agent and to optimize its potential application in LSIL treatment.

## 1. Introduction

Alpha lipoic acid (1,2-dithiolane-3-pentanoic acid, 1,2-dithiolane-3-valeric acid or 6,8-thioctic acid—ALA) has recently generated considerable clinical interest as a biologically active agent that can be effective in relieving symptoms related to numerous diseases (diabetes, age-related cardiovascular illness, metabolic obesity, etc.). ALA functions as the cofactor of oxidative decarboxylation reactions in glucose metabolism; the function that requires the disulfide group of the lipoic acid to be reduced to its dithiol form, dihydrolipoic acid, DHLA. It has been proven that the same process contributes to its efficiency as an antioxidant in biological systems—ALA has specific structural properties characterized by a dithiolane ring, enabling the existence of both the oxidized and reduced form that together create a potent redox couple that has a standard reduction potential of −0.32 V. This makes ALA a potent direct antioxidant capable of scavenging a variety of reactive oxygen species. Furthermore, it regenerates other antioxidants, chelates redox-active transition metals, and induces the uptake (or enhances the synthesis) of endogenous low molecular weight antioxidants or antioxidant enzymes [1]. Additionally, it has been proven to exert the wide range of anti-inflammatory activities, including the reduction in the lipopolysaccharide (LPS)-stimulated release of inflammatory cytokines, such as tumor necrosis factor alpha (TNF-α), interleukin-1 beta (IL-1β), IL-6, and LPS-induced expression of cyclooxygenase-2 (COX-2) and inducible nitric oxide synthase (iNOS) [2]. Recent investigation shows that by the combination of antioxidative and anti-inflammatory mechanisms, and through regulation of glucose homeostasis, ALA could also ameliorate lipid abnormalities in the hyper-lipemic and atherosclerotic environment in vivo [3,4].

The highest rates of clinical evidence exist for ALA’s application for improving the symptoms of peripheral neuropathy in patients with diabetes, in dosages ranging from 600 to 1800 mg per day [5]. It can positively affect the lipid profile in certain groups of patients, primarily by affecting increased low-density lipoprotein (LDL) levels [6]. Recent meta-analyses of clinical research also showed that supplementation with ALA for 2–48 weeks can modestly reduce body weight by 0.7–2.3 kg and body mass index (BMI) by up to 0.5 kg/m^2^ when compared with placebo in overweight individuals or patients with obesity [7]. Smaller studies indicate that ALA might be useful in the treatment of other diseases such as hemorrhoidal disease [8], Alzheimer disease [9] and male infertility [10]. 

A recent placebo-controlled study conducted by our research group proved the efficiency of a 3-month ALA supplementation (600 mg per day) in significantly inducing the rates of low-grade squamous intraepithelial lesion (LSIL) regression, and its effect has been attributed, in part, to the significant anti-inflammatory effects observed in the intervention group [11]. These results are particularly significant in the context of the general lack of therapeutic guidelines and scarce literature data on the effectiveness of nutritive intervention for patients with LSILs that might prevent progression of this condition into higher-grade SIL or cervical cancer.

The major hypothesis of this follow-up investigation was that the observed effectiveness of ALA in inducing LSIL regression can also be attributed to its antioxidant activity. Therefore, the effect of a 3-month supplementation with 600 mg of ALA on the parameters of antioxidant status was investigated in the same group of women diagnosed with LSILs. Additionally, observed responses to supplementation were discussed in relation to diet characteristics of the study participants, having in mind that the human antioxidant defense system resists modulation by dietary antioxidants and might depend on nutritional intake of antioxidants [12]. The secondary goal of the study was to investigate the impact of supplementation with ALA on lipid parameters of LSIL patients. Namely, ALA has been shown as a promising lipid lowering agent in patients diagnosed with diabetes type 2 or metabolic syndrome, while data on metabolically healthy patients are lacking. Obtained results would contribute to the limited knowledge of the possible modes of action of ALA in LSILs; the impact of dietary patterns on the effectiveness of supplementation with antioxidants (such as ALA); and the modes of the hypolipemic effect of ALA. 

## 2. Materials and Methods

### 2.1. Study Design

This study was registered at ClinicalTrials.gov (Clinical-Trials.gov, number NCT05485259) and was performed in accordance with the international, national and institutional guidelines pertaining to clinical studies and biodiversity rights, and it also complies with the CONSORT guidelines (Table S1, Figure S1). This study was approved by the Ethics Committee of the University of Zagreb, Faculty of Pharmacy and Biochemistry (no: 251-62-03-18-23) and the Ethics Committee of the University Clinical Centre Tuzla (no: 02-09/2-61-16). The progress of the study and potential side effects of ALA were monitored by the independent Data and Safety Monitoring Committee. Only the members of this committee were not blinded throughout the study and knew which group of patients received the treatment. 

This study was designed as a double-blind, randomized, placebo-controlled trial that recruited 100 female patients with the diagnosis of LSILs, which was determined after cytological screening, colposcopy and a targeted biopsy as described in detail previously [11]. 

Exclusion criteria were diabetes, malignant diseases, chronic inflammatory diseases, hysterectomy, abortion, destructive therapy of the cervix, HPV vaccination and menopause. Patients who reported regular use of dietary supplements and lipid-lowering pharmacotherapy were also not eligible for inclusion into the study. All recruited patients signed the informed consent (Table S1) for inclusion in the study. Recruitment of study participants was conducted at the University Clinical Centre Tuzla in the period between January 2020 and March 2022. 

The sample size was calculated by using a randomized clinical trial sample size formula (www.rikcalc.org/samplesize (accessed on 15 May 2019)): type α error was set at 5%; the study power was set at 85%; the ratio of the case to control was set to 1; and the expected dropout was 2%. The expected proportion of LSIL regression was set to 50% in the control group and in the treated group to 80%; the assumed dropout was 2%; and the superiority margin was set to 5%. Based on the used parameters, the total population study was determined to be 96 (48 subjects per group).

Block randomization was used to distribute participants to either the placebo or the intervention group in a 1:1 ratio. Patients were supplemented with either 600 mg per day of ALA (oral capsules containing all-rac ALA) or a placebo (provided as oral capsules containing rice starch, visually identical to ALA capsules) for 3 months. The capsules of both ALA and placebo were provided by Zada Pharmaceuticals (Lukavac, Bosnia and Herzegovina).

**Figure 1:**
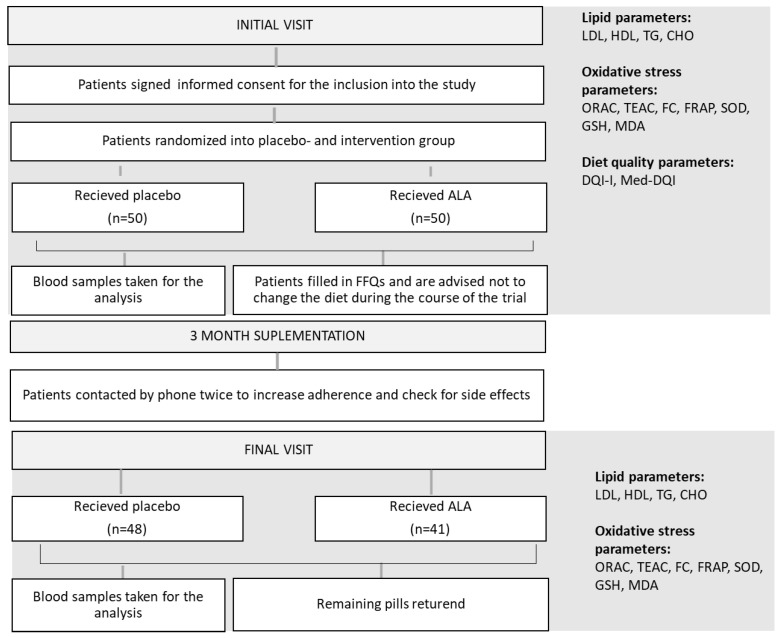
Flowchart of trial activities. ALA—alpha lipoic acid; LDL—low density lipoproteins; HDL—high density lipoproteins; TG—triglycerides; CHO—total cholesterol; ORAC—oxygen radical absorbance capacity; TEAC—Trolox equivalent antioxidant activity; FC—Folin–Ciocalteu reducing capacity; FRAP—ferric reducing activity; SOD—superoxide dismutase activity; GSH—reduced glutathione; MDA—malondialdehyde; DQI-I—Diet Quality Index-International; Med-DQI—Mediterranean Diet Quality Index; FFQ—Food Frequency Questionnaire.

The organization and the chronological order of trial activities are schematically presented in Figure 1.

At the initial appointment patients filled a standardized and validated semi-quantitative food questionnaire (FFQ) [13] with the help of trained staff to provide information on diet characteristics, use of dietary supplements, smoking, and physical activity. Through the course of the study, patients were contacted by telephone two times in order to improve the adherence and to check for the occurrence of any adverse effects. Three months after entry, participants were invited to the follow-up appointment.

At the initial and the follow-up appointment, blood samples were taken from the patients’ cubital vein, using the standard procedure. Biochemical parameters (total cholesterol (CHO), LDL, high density lipoproteins (HDL), triglycerides (TG) and oxidative status indicators (oxygen radical absorbance capacity (ORAC); Trolox equivalent antioxidant activity (TEAC); Folin–Ciocalteu reducing capacity (FC); ferric reducing activity (FRAP); superoxide dismutase (SOD) activity; reduced glutathione (GSH); and malondialdehyde (MDA) levels) were determined from the collected blood samples. Fasting venous blood was collected into a tube with serum separator gel at baseline and on the 90th day (BD Vacutainer, Becton Dickenson, NJ, USA). The serum was separated by centrifugation and multiple aliquots of each sample were either analyzed immediately (biochemical parameters) or stored at −80 °C for future analysis (oxidative stress parameters).

The capsules remaining after the 3-month supplementation period were to be returned for the adherence assessment. Patients were given advice not to change the level of physical activity or their eating habits throughout the course of the study.

### 2.2. Assessment of Biochemical Parameters

Biochemical parameters (CHO, LDL, HDL, and TG) were determined by standard laboratory procedures on an Architect ci8200 integrated system using the original reagents (Abbot Laboratories, Abbott Park, IL, USA).

### 2.3. Assessment of Oxidative Status Parameters

#### 2.3.1. Assessment of Total Antioxidant Capacity of Serum

Adequately diluted blood serums were used for all analyses. Spectrofluorometric and spectrophotometric measurements were conducted in 96-well plates using a Victor X3 plate reader (Perkin Elmer, Waltham, MA, USA). ORAC assay was conducted according to the method of Ou and co-workers [14], which measures free radical oxidation of a fluorescent probe through the change in its fluorescence intensity. It is run to completion and the dynamic change in fluorescence of the probe over time is accounted for by calculating the area under the fluorescence decay curve (AUC). It compares the extent of the fluorescence quenching induced by the sample/standard/blank solution and considers both the inhibition degree and inhibition time, which is considered a methodological improvement. Fluorescence measurements were made at an excitation wavelength of 485 nm and an emission wavelength of 530 nm. Trolox was used for designing the calibration curves by plotting the AUC with corresponding Trolox concentrations. The results were expressed as mg L^−1^ of Trolox equivalents (TEs). A TEAC assay was performed according to the procedure of Re and co-workers [15], which measures the decrease in the absorbance of a preformed ABTS^+^ solution in the presence of an antioxidant compound. The ABTS^+^ chromophore (blue/green) is generated through the reaction between ABTS and potassium persulfate. The decolorization of ABTS^∙+,^ in the presence of an antioxidant, can be measured at 734 nm, and the results were expressed as mg L^−1^ TEs. The FC reducing capacity of the serum was determined according to the method of Ainswort and Gillespie [16], which relies on the transfer of electrons in alkaline medium from phenolic compounds to phosphomolybdic/phosphotungstic acid complexes, which are determined spectroscopically at 765 nm. Results were expressed as mg L^−1^ of gallic acid equivalents (GAE). The FRAP method [17] measures ferric to ferrous ion reduction in the presence of antioxidants at low pH, causing the formation of a ferrous-tripyridyl-triazine complex that can be measured spectrophotometrically at 593 nm. The obtained results are expressed as µmol L^−1^ TEs. 

#### 2.3.2. Assessment of the Activity of the Endogenous Antioxidant System

GSH was determined by the modified method of Machado and Soares [18], which measures the formation of the fluorescent complex between monochlorbimane and GSH that can be monitored at an excitation wavelength of 355 nm and an emission wavelength of 460 nm. Results were expressed as µmol L^−1^. SOD activity was measured by using a 19160 SOD determination kit (Sigma-Aldrich St. Louis, MI, USA). This method measures the inhibition activity of SOD that can be monitored as a decrease in absorbance of a water-soluble formazan dye formed in the presence of the superoxide anion at 440 nm. Relative SOD activity is presented as % of absorbance inhibition (measured at 440 nm).

#### 2.3.3. Assessment of Lipid Peroxidation

The determination of MDA was based on the method by [19] with a few modifications. The method is based on the reaction of thiobarbituric acid (TBA) and MDA. MDA under acidic conditions reacts with TBA in a ratio of 1:2, whereby a red pigment is formed, the absorbance of which is measured at 532 nm. 1,1,3,3 tetraethoxypropane has been used as the standard for the preparation of the calibration curve, and the obtained results were expressed as µmol L^−1^. 

### 2.4. Analysis of Dietary Characteristics and Calculation of Diet Quality Indexes

Semiquantitative FFQ used for the assessment of dietary characteristics was designed as a 192-item questionnaire with one month as the reference period of intake. It included questions on the frequency of consumption and the approximate amount of 100 dietary items listed, and additional queries about supplementation used and eating habits. The questionnaire was constructed as a modification of a previously published questionnaire [20] regarding serving sizes and the national specificity of foods. The study participants filled out the FFQ (with the help of a research team member if necessary). Average daily food intake was calculated for each participant based on the data reported in the FFQ. Dietary intake for selected nutrients was calculated using national food composition tables [21] and serving sizes according to USDA Dietary Guidelines for Americans, 2020–2025 [22].

The quality of dietary habits of participants was estimated by the calculation of specific indexes: the Diet Quality Index-International (DQI-I) that serves as a composite measure of general diet quality created to evaluate the healthfulness of a diet; and an index evaluating the compatibility of dietary habits to the concepts of the Mediterranean diet: Mediterranean Diet Quality Index (Med-DQI). For the DQI-I the defined score range is 0–100, where 100 indicates the highest quality of nutrition. The DQI-I focuses on four quality characteristics of a diet: variety (overall food group variety and within-group variety for protein), adequacy (vegetable, fruit and grain group; dietary fibre, protein, Fe, Ca and vitamin C), moderation (total fat, saturated fat, cholesterol, sodium and empty calorie foods) and overall balance (macronutrient ratio and fatty acid ratio) [23].

For the calculation of this index olive oil was added as particular category (with a score increasing with a lower intake, as opposed to cholesterol or saturated fat intake). The Protein intake category is divided into meat and fish intake (with intake of fish having an opposite gradient to meat). For the Med-DQI, every nutrient or food group is assigned three scores (0, 1 and 2) depending on either recommended guidelines or (where there was no specific recommendation for item) by dividing the population’s consumption into tertials. The total Med-DQI for each subject is calculated by summing all scores. The best Med-DQI has a score of 0. Scores between 1 and 4 were considered as good; scores between 5 and 7 as medium to good; scores between 8 and 10 as under medium to poor; and scores between 11 and 13 as poor [24,25]. 

### 2.5. Statistical Analysis

GraphPad Prism Version 8.4.3 (GraphPad Software LLC, San Diego, CA, USA) and MedCalc statistical software ver.14.8.1.0. (MedCalc Software Lcd., Ostend, Belgium) were used for statistical analysis. Results were presented as medians and interquartile ranges. For numerical variables nonparametric statistical tests were used (since the number of patients per group was rather low (*n* ≤ 50)). To identify between-group differences either a Wilcoxon’s paired-rank test or a Mann–Whitney’s test was used. Comparison of binary outcomes was made by 2 × 2 tabulation and a risk ratio (RR), 95% confidence intervals (CIs), and *p*-values were calculated.

## 3. Results

### 3.1. The Baseline Characteristics of the Participants

A total of 100 female patients were included to this study and were allocated to placebo and intervention group in a 1:1 ratio. A total of 41 participants in the intervention group and 48 participants in the placebo group finished the study; the remaining patients (*n* = 11) reported that they did not finish the study due to personal reasons.

The baseline characteristics of the study groups are presented in Table 1. Different lifestyle and selected diet characteristics that might have an impact on the antioxidant status of the participants were compared between the two arms. A complete list of diet characteristics of the study participants assessed by FFQ analysis is presented in Table S2.

As presented in Table 1, there were no significant differences in baseline characteristics or diet between the placebo and intervention groups. The age of participants, and the percentage of smokers in the two study groups were similar (*p* = 0.082 and *p* = 0.332, respectively). The percentage of smokers in the placebo and the intervention group was 29.2% and 19.5%, respectively). Compliance was assessed based on the number of returned unused capsules after a 3-month supplementation period and was high in both groups (80.6% and 84.4%, respectively).

Dietary characteristics relevant for antioxidant status and lipid profile were compared between the two groups of patients: intake of fruit and vegetables, vitamin C, vitamin E and carotenoids (as antioxidant, protective factors); intake of energy, meat, red meat, animal protein, saturated fat, unsaturated fat, and cholesterol (as factors that could possibly contribute to oxidative stress and dyslipidemia). General compliance of participants’ dietary habits with general dietary guidelines or principles of the Mediterranean diet (quantified and expressed as the DQI-I and Med-DQI, respectively) has been compared between the groups. The analysis showed that there were no statistically significant differences between the placebo and intervention groups regarding any of the observed diet characteristics. Additional details on the intake of nutrients of study participants are presented in Table S2.

### 3.2. Impact of ALA Supplementation on Oxidative and Lipid Status Parameters

Impact of ALA supplementation on oxidative status parameters has been investigated previously in diabetic patients [26,27], patients with multiple sclerosis [28] and non-alcoholic liver disease [29] and patients on hemodialysis [30,31]. Obtained results varied significantly, depending on the oxidative stress marker and the significance of the observed effect (some studies showed significant impact of supplementation, others did not). 

This investigation focused on serum total antioxidant capacity (TEAC, ORAC, FRAP and FC reducing potential), activity of antioxidant enzymes (SOD), status of endogenous antioxidants (GSH) and lipid peroxidation markers (MDA) of patients with LSILs after 3 months of ALA supplementation. Values between the placebo and the treated groups were compared at the initial visit and the 3-month follow-up visit and are presented in Table 2. The differences between the placebo and the treated group at the initial and the 3-month follow-up visits are presented in Table 3. 

Changes in other anthropometric and biochemical parameters in the placebo and intervention groups are presented in Table S3.

As presented in Table 2, at the initial measurement there were no significant differences between medians of MDA levels (*p* = 0.151), FRAP (*p* = 0.381), SOD (*p* = 0.180), ORAC (*p* = 0.488), or GSH (*p* = 0.389) in the placebo and the intervention groups; TEAC was significantly higher (*p* = 0.048) and FC reducing capacity was significantly lower in the intervention group (*p* = 0.005). After the 3 months of supplementation the situation remained unchanged except for the GSH levels that were now significantly lower in the intervention group compared to the placebo (*p* = 0.050). However, the apparent decrease of GSH values that was observed in the ALA-supplemented group after 3 months of supplementation was found to be statistically insignificant (*p* = 0.411). Moreover, as presented in Table 3, none of the monitored oxidative status parameters were significantly changed, neither in the placebo nor in the intervention groups.

Diet characteristics can play a significant role in modulating the overall effectiveness of antioxidant supplements [32] by influencing the baseline host antioxidant status or through specific nutrient interactions—influencing the bioavailability of supplemental antioxidants or resulting in synergistic/antagonistic reactions. Therefore, a subgroup analysis was conducted to compare the ALA effectiveness in subgroups of patients with high (>11) and low (<7) Med-DQI (indicating low and high degree of adherence to Mediterranean dietary patterns). The Med-DQI was chosen because it quantifies the levels of adherence to the Mediterranean diet and dietary diversity, which are known to contribute to lower antioxidant and inflammation biomarkers [33,34,35].

**Figure 2:**
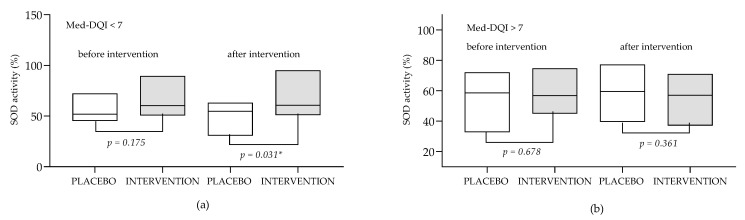
SOD activity in the subgroups of patients with Med-DQI < 7 (**a**) and Med-DQI > 7 (**b**) before and after ALA supplementation. Data are presented as medians (min to max values). Tested by Mann–Whitney test. * The observed difference is statistically significant (*p* < 0.05). SOD—superoxide dismutase activity; Med-DQI—Mediterranean Diet Quality Index.

Subgroup analysis showed that the effects of antioxidant supplementation on oxidative status biomarkers might be significantly affected by the patient’s dietary characteristics. As presented in Figure 2, in the subgroup of patients with higher levels of compatibility with Mediterranean dietary patterns (Med-DQI < 7) supplementation with ALA showed positive effects on SOD activity (it prevented the decrease in SOD activity that was observed in the placebo group). This was not observed in the in the remaining patients (Med-DQI > 7).

Impact of ALA on lipid parameters has been investigated to some extent with most available studies focused on patients with metabolic diseases showing that ALA might exert mild lipid lowering properties [6]. Considering ALA could provide clinically significant benefits in other indications not primarily associated with disturbances of the lipid profile (such as LSILs), it is of great importance to investigate its effect on lipid parameters in metabolically healthy patients. To investigate the effect of ALA supplementation on lipid parameters, LDL, HDL, TG and CHO values were investigated (Table 2 and Table 3). 

As presented in Table 2, initially there were no significant differences in lipid parameters between the placebo and intervention groups. However, after 3 months, the supplementation LDL levels in the intervention group were significantly higher compared to the placebo group (*p* = 0.033). Table 3 shows that during the 3-month supplementation period, CHO and LDL levels of the placebo group remained unchanged, while in the intervention group they increased from 5.19 to 5.69 (*p* = 0.001) and from 2.89 to 3.46 (*p* = 0.006). The obtained results are not consistent with the results of other authors who showed that ALA either decreased LDL or showed no significant effect [6]; however, those data were mostly obtained in studies focusing on patients with metabolic diseases and hyperlipidemia. 

**Figure 3:**
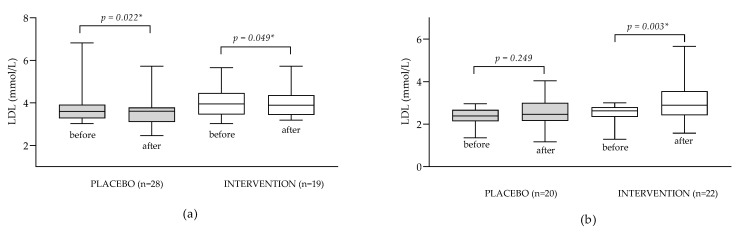
Impact of ALA supplementation on LDL status in subgroups of patients with hyperlipidemia (LDL > 3 mmol L^−1^) (**a**); and normolipemic patients (**b**). Data are presented as medians (min to max values). Tested by Wilcoxon’s paired-rank test. * Difference is statistically significant (*p* < 0.05).

To test our hypothesis that ALA could have different effects on LDL levels depending on the initial lipid status, we conducted subgroup analyses focusing specifically on participants with hypercholesterolemia (LDL > 3 mmol L^−1^) and specifically on normolipemic patients (LDL ≤ 3.00 mmol L^−1^). The obtained results are presented in Figure 3. 

In patients with hyperlipidemia supplementation with ALA resulted in a very small but statistically significant decrease in LDL values (the median decreased from 3.95 to 3.89 mmol L^−1^; *p* = 0.049); however, a more pronounced effect has been observed in the placebo group (with a decrease from 3.76 to 3.54 mmol L^−1^; *p* = 0.022). This might be explained by a possible positive modification of dietary and lifestyle habits of the patients upon recruitment into the dietary clinical study (despite clear instruction that dietary habits and lifestyle should not be changed during the study), which has been noted in the literature [36]. In the subgroup of patients with normal LDL levels, a significant increase in LDL levels was observed in the intervention but not in the placebo group (median increased from 4.01 to 4.07 mmol L^−1^; *p* = 0.003), which confirms our hypothesis that the effect of ALA supplementation on lipid status might be affected by the initial lipid status of the patient. 

## 4. Discussion

### 4.1. Dietary Characteristics of Participants

Patient response to antioxidant supplementation is in great part conditioned by his general dietary habits and nutritional intake of antioxidants. For this purpose, it was important to investigate if dietary habits of the participants included in this study are comparable to the general population. As presented in Table S1, participants’ diets were characterized with high median energy intake (3160 kcal), high medians of fat and saturated fat intake (186 g and 62.2 g, respectively), low medians of polyunsaturated fatty acid (PUFA)/saturated fatty acid (SF) and monounsaturated fatty acid (MUFA)/SF ratio (0.85 and 0.93, respectively). The intake of red meat was in line with recommendations (2.92 portions per week), but the intake of fish, fruits and vegetables were significantly lower, particularly for fish (0.54, 3.54 and 3.71 servings per week, respectively). Generally, it can be concluded that the participants of this study consumed a Western-type diet characterized with low DQI-I and Med-DQI (63.5 (out of 100) and 9 (categorized as medium to poor), respectively). This was reflected in the low intake of vitamins and minerals. While the intake of vitamin C, B-complex (except folate), vitamin E and vitamin A were above recommendations in >75% of patients, intake of vitamin D and folate was problematic and adequate intake was observed in only 3% and 38% of study participants, respectively. Among minerals, intake of Fe and Ca was particularly problematic and only a minority of patients had adequate intake (35% and 42%, respectively). Intake of sodium was higher than recommended in 28% of participants and intake of potassium was lower than recommended in 70% of participants.

Because this study has been conducted on women only, and due to the lack of precise data for nutritional habits in Bosnia and Herzegovina or surrounding countries, it was hard to assess if dietary habits of study participants are comparable to average habits of the general population. The obtained data were therefore compared to the dietary habits of women in eastern and central European countries, which were investigated previously in the frameworks of the HAPIEE study [37]. General dietary characteristics regarding nutritional quality, food group, energy and average micronutrient intakes were comparable; intake of fat, saturated fat and dietary fibers were somewhat higher in our study.

### 4.2. Impact of ALA Supplementation on Oxidative Status Parameters

Even though ALA has been recognized primarily as a potent antioxidant that exerts its action through different various direct and indirect mechanisms [1], our results showed that its application did not result in the significant change in any of the observed oxidative status parameters.

Clinical data obtained by other authors are scarce and inconsistent. Derosa and co-authors [26] showed that supplements containing 600 mg of ALA significantly reduced MDA levels and increased activities of SOD and GSH levels in diabetic patients after 3 months of supplementation. They observed a decrease in inflammation markers in the intervention group which is consistent with our previously published results [11]. Khalili and co-authors [27] found that SOD activity, glutathione peroxidase activity and MDA levels were not affected by consumption of 1200 mg of ALA for 3 months in patients with multiple sclerosis; however, TEAC values were positively affected. In patients with non-alcoholic liver disease 3 months of supplementation with 1200 mg of ALA significantly decreased MDA levels and increased the total antioxidant capacity of serum while other oxidative status parameters remained unaffected by ALA supplementation [28]. In patients with renal disease on hemodialysis, supplementation with ALA did not affect oxidative status parameters [30,31]. In the pilot study by Huang and Gitelman [27], ALA was not an effective treatment for decreasing oxidative damage in adolescents with type 1 diabetes.

Inconsistencies in results obtained by various authors can be explained, in part, by differences in baseline characteristics of participants—pathologies, metabolic status, age, sex, supplementation protocols (600 mg vs. 1200 mg) or composition of tested supplements. For example, Derosa and co-workers [26] tested a multicomponent formulation, none of the studies included information about ALA isomer ratios in the tested formulations, etc.

Additionally, this study has been conducted exclusively on women which might have influenced the obtained results. Recent publications [38,39,40] have shown that the steady state redox status can differ significantly between men and women, showing higher antioxidant status in women, at least partially because of estrogen, even though the impact of other factors, such as age, diet, or smoking, seem to be of greater relevance. The impact of gender on the response to antioxidant supplementation has been investigated in two smaller studies indicating no significant differences in the responses between men and women [40,41].

It is important to emphasize that even though diet plays a significant role in modulating the overall effectiveness of antioxidant supplements [32], none of the above-mentioned studies investigated the diet characteristics of the participants and took those data into account when discussing study results. It was our assumption that the most important aspect of diet to be associated with the patient’s response to antioxidant supplementation would be dietary intake of antioxidants, and the diet quality index that primarily considers this aspect of nutrition is the Med-DQI. Results of the conducted subgroup analysis based on the Med-DQI showed that supplementation with ALA positively affected SOD activity (Figure 2). This observation can be explained by the fact that ALA increases the gene expression of the primary antioxidant enzymes such as superoxide dismutase (SOD) which can be monitored as the increase in activity in the serum [42]. In our study the effect was visible only in the subgroup of female patients with a high degree of adherence to a Mediterranean-type diet. A possible explanation of the obtained results is that in the state of higher oxidative stress increased SOD expression does not result in increased serum activity because SOD is being extensively used in cellular oxidative reactions. Contrary to that, high dietary intake of other antioxidants that reduce the formation of ROS (including superoxide anion) decreases the depletion of SOD so that the effect of ALA on SOD serum activity is visible and significant.

### 4.3. Impact of ALA Supplementation on Lipid Parameters

The obtained results pointing out a mild hyperlipidemic effect of ALA supplementation are partially inconsistent with the results obtained by other authors. Lipid-reducing properties of ALA have been proven in numerous studies, but due to heterogenicity of the studied populations and dosing regimens (400–1200 mg/day) it is hard to estimate its actual clinical effectiveness in hypercholesterolemia. A meta-analysis by Haghighatdoost and Hariri [6] showed that ALA may significantly decrease total CHO, LDL and TG levels but that the results obtained so far are contradictory and more research is needed to draw more specific conclusions. A more recent meta-analysis [43] found that the observed effects of ALA in diabetic patients were not clinically significant.

As mentioned previously, most of the available research has been focused on patients with metabolic diseases with pronounced dyslipidemia while our study focused on metabolically healthy patients which might have contributed to the observed lack of a hypocholesterolemic effect of ALA. The conducted subgroup analysis (Figure 3) revealed that the effect of ALA supplementation in patients with hyperlipidemia causes a slight, but statistically significant, decrease in LDL levels which is consistent with the results of other studies [6]. On the contrary, supplementation of patients with normal LDL levels led to significantly increased LDL levels, which has not been shown before (to our knowledge).

Additionally, since this study was conducted in exclusively female patients, gender-related differences might have contributed to differences of our results compared to the other available research. Namely, to our knowledge, the impact of ALA on lipid parameters has not been investigated in exclusively female patients. According to Ordovas and co-workers [44] women may be less responsive to dietary intervention in terms of LDL cholesterol lowering. Additionally, men and women respond differently to statin therapy and the observed effects have not been thoroughly investigated. They are assumed to include multiple mechanism differences in therapy adherence, gene polymorphisms or hormonal differences [45,46] that might also be involved in the regulation of responses to ALA supplementation.

The observed effect of ALA supplementation is related to its mechanism of lipid-lowering action which has not been totally elucidated. The mechanism is thought to be multifactorial—ALA is probably capable of initiating LDL receptor synthesis in the liver which in turn increases the uptake of cholesterol back to the hepatic system and increases synthesis of apoprotein A component [47]. A possible impact on lipoprotein lipase activity and inhibition of HMG-CoA reductase have also been suggested [48,49]. It is possible to hypothesize that the lipid-lowering effects of ALA can only be manifested in patients with a certain level of disfunction regarding the lipid metabolism and that it depends on the initial lipid status of the patients. This hypothesis is partially backed up by the conclusions of several clinical studies conducted in normolipemic patients or patients with a lower degree dyslipidemia. For example, Gosselin and co-authors [50] showed that ALA is not effective in modulating serum lipids in prediabetic patients, and Iannuzzo and co-authors [51] showed that ALA had no effect on body weight and blood lipid levels in (mainly normolipemic) schizophrenic subjects. Further studies are necessary to elucidate the exact mechanisms of the lipid-lowering effects of ALA and the importance of initial lipid status and dosing regimen on the clinical effects of supplementation.

## 5. Conclusions

The results of the conducted trial demonstrate that monitored oxidative stress biomarkers of patients with a LSIL diagnosis and adherence to a typical western-style diet are not significantly affected by 3 months of supplementation with 600 mg of ALA. Subgroup analysis showed that the impact of ALA supplementation on oxidative status parameters might be significantly affected by the diet quality of the participants, particularly by the degree of compliance to a Mediterranean dietary pattern. Unexpectedly, ALA supplementation resulted with a small but statistically significant increase in LDL and CHO levels, indicating that the lipid-lowering effect of ALA observed in some studies might depend on the initial lipid status of the participants (which has been confirmed by post-hoc subgroup analysis).

The obtained results contribute significantly to the current knowledge on the possibilities and limitations of the utilization of ALA as a dietary supplement and provide additional insight into the possible mechanisms of actions. Larger studies are necessary, that will enable a more comprehensive investigation of significant confounding factors. Additionally, having in mind the relatively low and variable bioavailability of ALA, the impact of supplementation of ALA levels/status should be monitored and considered when interpreting the obtained data.

## Figures and Tables

**Table 1:** Baseline characteristics of placebo and intervention groups.

	Placebo Group (*n* = 48)	Intervention Group (*n* = 41)	*p*
	Lifestyle characteristics		
*Age (years)*	*37 (28–46)*	*43 (34–47)*	*0.082*
Cigarette smoking * *(cigarettes per day)*	*14*	*8*	*0.333*
Compliance * *(returned tablets)*	*35*	*28*	*0.405*
	Diet characteristics **		
*Energy (kcal)*	*3104 (1937–4249)*	*3530 (2015–4404)*	*0.374*
*Fruits (servings per week)*	*2.78 (1.90–5.45)*	*3.66 (1.56–5.88)*	*0.365*
*Vegetables (servings per week)*	*3.49 (2.21–4.67)*	*4.17 (2.83–5.86)*	*0.252*
*Animal protein (g/day)*	*40.61 (24.33–62.90)*	*36.05 (23.29–54.07)*	*0.315*
*Meat (servings per week)*	*3.95 (2.25–7.37)*	*4.20 (2.54–7.24)*	*0.558*
Red meat (servings per week)	2.66 (0.00–14.36)	4.05 (0.00–33.76)	0.852
Fat (g)	167.9 (99.7–252.5)	190.6 (103.6–293.8)	0.176
Saturated fat (g)	53.4 (29.6–85.5)	69.75 (34.26–103.7)	0.202
Cholesterol (mg)	290.4 (178.3–473.4)	291.6 (188.8–422.4)	0.809
Vitamin C (mg)	199.3 (139.0–302.4)	175.7 (95.91–321.1)	0.570
Vitamin E (mg)	21.44 (12.5–44.4)	33.61 (17.17–48.06)	0.262
Carotenoids (mg) ^#^	7.58 (4.54–15.0)	14.43 (6.363–18.65)	0.064
DQI-I ***	63.55 (57.4–67.7)	63.64 (55.00–68.79)	0.308
Med-DQI ***	9.00 (7.25–10.0)	9.00 (7.00–10.00)	0.516

Results are expressed as the medians (interquartile range); tested by Wilcoxon’s paired-rank test. * Results are expressed as numbers; tested by Fisher’s exact test. ** Results are expressed as average daily intake. ^#^ Sum of beta carotene, Lutein, zeaxanthin and lycopene. *** Calculated using the data obtained by semiquantitative FFQ. DQI-I: Diet Quality Index-International; Med-DQI: Mediterranean Diet Quality Index. Part of the presented data that has been previously published [11] is written in italics.

**Table 2:** Study outcomes at the initial visit and the 3-month follow-up (placebo vs. intervention).

	Placebo (*n* = 48)	Intervention (*n* = 41)	*p* *	Placebo (*n* = 48)	Intervention (*n* = 41)	*p* **
	initial measurement			3-month follow-up measurement		
MDA (µmol L^−1^)	0.566 (0.373–0.819)	0.604 (0.444–1.011)	0.151	0.546 (0.346–1.072)	0.617 (0.462–1.13)	0.472
FRAP (µmol L^−1^ TE)	395.5 (336.2–445.1)	398.3 (360.9–449.1)	0.381	403.9 (345.0–462.8)	392.7 (374.6–428.8)	0.768
SOD (inhibition (%))	58.35 (51.64–64.16)	59.21 (54.23–66.31)	0.180	59.45 (52.15–63.89)	59.43 (53.15–64.12)	0.348
ORAC (mg L^−1^ TE)	5471 (4583–6295)	4741 (3600–6447)	0.488	4759 (3978–6310)	5222 (4145–6119)	0.184
TEAC (mg L^−1^ TE)	296.7 (272.1–328.5)	322.7 (282.5–352.8)	0.048	299.7 (273.4–326.0)	321.9 (298.3–351.0)	0.003
FC (mg L^−1^GAE)	1316 (1181–1426)	1028 (697.3–1289)	0.005	1310 (1164–1454)	1157 (710.3–1291)	0.047
GSH (µmol L^−1^)	48.33 (44.54–54.60)	47.19 (44.21–51.63)	0.389	48.46 (45.37–54.29)	45.72 (42.04–50.57)	0.050
CHO (mmol L^−1^)	5.295 (4.658–6.110)	5.190 (4.680–6.220)	0.502	5.240 (4.753–6.083)	5.690 (5.225–6.650)	0.057
LDL (mmol L^−1^)	3.160 (2.473–3.700)	2.890 (2.600–3.895)	0.712	3.115 (2.543–3.668)	3.460 (2.840–4.080)	0.033
HDL (mmol L^−1^)	1.400 (1.270–1.633)	1.420 (1.185–1.740)	0.941	1.435 (1.200–1.620)	1.450 (1.235–1.890)	0.118
TG (mmol L^−1^)	1.120 (0.850–1.785)	1.260 (0.795–1.945)	0.320	1.040 (0.843–1.750)	1.180 (0.820–2.050)	0.402

Results are expressed as medians (interquartile range). Tested by Wilcoxon’s paired-rank test. * Comparing placebo and treated group of patients at initial visit. ** Comparing placebo and treated group of patients at the 3-month follow-up visit. MDA—malondialdehyde content; FRAP—ferric reducing power; SOD—superoxide dismutase activity; ORAC—oxygen radical absorbance capacity; TEAC—Trolox equivalent antioxidant activity; FC—Folin–Ciocalteu reducing capacity; GSH—reduced glutathione; CHO—total cholesterol; LDL—low-density lipoproteins; HDL—high density lipoproteins; and TG—triglycerides.

**Table 3:** Changes in study outcomes between the initial and the 3-month follow-up appointments.

	Placebo Initial	Placebo 3-Month Follow-Up	*p* *	Intervention Initial	Intervention 3-Month Follow-Up	*p* **
MDA (µmol L^−1^)	0.566 (0.373–0.819)	0.546 (0.346–1.072)	0.420	0.604 (0.444–1.011)	0.617 (0.462–1.13)	0.327
FRAP (µmol L^−1^ TE)	395.5 (336.2–445.1)	403.9 (345.0–462.8)	0.207	398.3 (360.9–449.1)	392.7 (374.6–428.8)	0.972
SOD (inhibition (%))	58.35 (51.64–64.16)	59.45 (52.15–63.89)	0.875	59.21 (54.23–66.31)	59.43 (53.15–64.12)	0.291
ORAC (mg L^−1^ TE)	5471 (4583–6295)	4759 (3978–6310)	0.264	4741 (3600–6447)	5222 (4145–6119)	0.226
TEAC (mg L^−1^ TE)	296.7 (272.1–328.5)	299.7 (273.4–326.0)	0.593	322.7 (282.5–352.8)	321.9 (298.3–351.0)	0.581
FC (mg L^−1^ GAE)	1316 (1181–1426)	1310 (1164–1454)	0.996	1028 (697.3–1289)	1157 (710.3–1291)	0.888
GSH (µmol L^−1^)	48.33 (44.54–54.60)	48.46 (45.37–54.29)	0.703	47.19 (44.21–51.63)	45.72 (42.04–50.57)	0.411
CHO (mmol L^−1^)	5.295 (4.658–6.110)	5.240 (4.753–6.083)	0.941	5.190 (4.680–6.220)	5.690 (5.225–6.650)	0.001
LDL (mmol L^−1^)	3.160 (2.473–3.700)	3.115 (2.543–3.668)	0.277	2.890 (2.600–3.895)	3.460 (2.840–4.080)	0.006
HDL (mmol L^−1^)	1.400 (1.270–1.633)	1.435 (1.200–1.620)	0.020	1.420 (1.185–1.740)	1.450 (1.235–1.890)	0.002
TGC (mmol L^−1^)	1.120 (0.850–1.785)	1.040 (0.843–1.750)	0.301	1.260 (0.795–1.945)	1.180 (0.820–2.050)	0.447

Results are expressed as medians (interquartile range). Tested by Wilcoxon’s paired-rank test. * Comparing placebo at initial visit and after a 3-month treatment. ** Comparing treated group at initial visit and after a 3-month treatment. MDA—malondialdehyde content; FRAP—ferric reducing power; SOD—superoxide dismutase activity; ORAC—oxygen radical absorbance capacity; TEAC—Trolox equivalent antioxidant activity; FC—Folin–Ciocalteu reducing capacity; GSH—reduced glutathione; CHO—total cholesterol; LDL—low-density lipoproteins; HDL—high density lipoproteins; and TG—triglycerides.

## Data Availability

The data that support the findings of this study are available from the corresponding author (D.V.Č.) upon reasonable request.

## Supplementary Materials

The following supporting information can be downloaded at: https://www.mdpi.com/article/10.3390/antiox12091670/s1. Figure S1: CONSORT 2010 Flow Diagram; Table S1: CONSORT 2010 checklist of information to include when reporting a randomized trial*; Table S2: Diet characteristics of study participants as assessed by semiquantitative Food Frequency Questionnaire (FFQ); Table S3: Changes in other anthropological and biochemical markers between the initial and the 3-month follow-up appointment in placebo and intervention groups.
