# Supplementary material for: Effect of Alpha Lipoic Acid Supplementation on Oxidative Stress and Lipid Parameters in Women Diagnosed with Low-Grade Squamous Intraepithelial Lesions (LSILs): A Double-Blind, Randomized, Placebo-Controlled Trial
Source: Antioxidants (Basel). 2023 Aug 25;12(9):1670. doi: 10.3390/antiox12091670 (PMC10525309; doi:10.3390/antiox12091670)
Supplement: Supplementary file 1 [file antioxidants-12-01670-s001.zip › Table S2.pdf]

Table S2. Diet characteristics of study participants as assessed by semiquantitative food frequency questionary (FFQ)

Average daily intake	Median	Min	Max
Water (g)	1855	466	6409
Energy (kcal)	3160	586	8487
Protein (g)	81,8	20,8	416,1
AMDR protein (%)	11,3	1,7	24,3
Fat (g)	186,0	24,3	1695
AMDR fat (%)	53,7	25,7	94,6
SF) (g)	62,2	8,0	377,6
AMDR saturated fat (%)	17,01	9,36	32,74
MUFA (g)	59,9	10	791,2
PUFA (g)	48,07	3,76	479,0
PUFA/SF ratio	0,85	0,16	2,6
MUFA/SF ratio	0,93	0,50	4,74
Cholesterol (mg)	299,8	51,8	1402
Carbohydrates (g)	297,2	55,8	781,0
Dietary fibre (g)	32,08	5,19	141,1
Vitamin A (μg RAE)	773,4	70,8	4896
Retinol (μg)	404,7	18,1	2834
β-carotene (μg)	2552	23,0	12599
Lycopene (μg)	3524	0,00	111585
Lutein + zeaxanthin (μg)	3612	117,1	20094
Vitamin D (μg)	3,6	0,25	44,4
Vitamin E (mg)	29,16	1,41	353,4
Vitamin K (μg)	193,9	21,1	1516
Thiamine-B1 (mg)	1,72	0,41	5,41
Riboflavin-B2 (mg)	2,35	0,47	9,82
Niacin-B3 (mg)	23,4	6,45	85,4
Pantothenic acid-B5 (mg)	6,48	1,37	24,9
Pyridoxine-B6 (mg)	2,15	0,32	6,51
Folate-B9 (μg)	377	70,8	2017
Choline (mg)	342,3	55,9	1398
Cobalamins-B12 (μg)	4,71	0,56	23,8
Vitamin C	192,2	11,5	1496
Calcium (mg)	958,5	184,9	6468
Iron (mg)	16,4	4,15	77,8
Magnesium (mg)	430,5	96,9	1575
Phosphorus (mg)	1383	382,2	5934
Zinc (mg)	10,74	2,88	39,7
Copper (mg)	1,84	0,43	10,0
Manganese (mg)	4,15	0,54	12,2
Selenium (μg)	95,9	19,2	542,2
Potassium (mg)	4070	941	13336
Sodium (mg)	1757	311,0	11879
**Weekly intake**	**median**	**min**	**max**
Fruit (serving)	3,54	0,06	18,9
Vegetable (serving)	3,71	0,26	23,9

AMDR-acceptable macronutrient distribution ranges; SF-saturated fat; PUFA-polyunsatturated fatty acid; MUFA-monounsaturated fatty acid; RAE-retinol activity equivalents
