# Supplementary material for: Effect of Alpha Lipoic Acid Supplementation on Oxidative Stress and Lipid Parameters in Women Diagnosed with Low-Grade Squamous Intraepithelial Lesions (LSILs): A Double-Blind, Randomized, Placebo-Controlled Trial
Source: Antioxidants (Basel). 2023 Aug 25;12(9):1670. doi: 10.3390/antiox12091670 (PMC10525309; doi:10.3390/antiox12091670)
Supplement: Supplementary file 1 [file antioxidants-12-01670-s001.zip › Table S3.pdf]

Table S3. Changes of other anthropological and biochemical markers between the initial and the 3-month follow-up appointment in placebo- and intervention group.

	**placebo initial**	**placebo 3-month follow up**	**P***	**intervention initial**	**intervention 3-month follow-up**	**P****
[1]**body mass (kg)**	59 (48-76)	57 (50-71)	0.353	63 (49-81)	60 (49-77)	0.429
[1]**BMI (kg/m^2)**	25.0 (21.6-27.1)	24.8 (21.9-27.0)	0.727	23.9 (21.6-26.9)	23.6 (21.4-27.1)	0,613
[1]**hsCRP*** **(mg/L)**	0.86 (0.47-1.32)	1.11 (0.61-1.98)	<0.001	1.77 (0.82-2.44)	0.89 (0.39-1.68)	<0.001
[1]**FI*** **(g/L)**	3.30 (2.4-3.9)	3.7 (2.7-4.5)	<0.001	3.9 (3.1-4.4)	3.3 (2.6-3.8)	<0.001
[1]**SE*** **(mm/h)**	16 (12-23)	20 (16-26)	<0.001	20 (15-26)	16 (12-22)	<0.001
	**increased**			**decreased**		
	**placebo**	**intervention**		placebo	**intervention**	
**IL6**	3	0		0	11	

Results are expressed as medians (interquartile range) [1]Tested by the Mann-Whitney's test; comparing the values in the initial and the 3-month follow-up visit in placebo treated group. *data adopted from Divković and co-authors [10].
